# Clinicopathological and immunohistochemical description of an intrapelvic hepatoid gland carcinoma in a 14-year-old Teckel dog

**DOI:** 10.1080/01652176.2017.1404167

**Published:** 2017-12-07

**Authors:** Joelson Jardim, Priscila E. Kobayashi, Patrícia D. Cosentino, Ana Alcaraz, Renée Laufer-Amorim, Carlos E. Fonseca-Alves

**Affiliations:** a Saúde Animal Veterinary Hospital, Sorocaba, Brazil; b Department of Veterinary Clinic, School of Veterinary Medicine and Animal Science, São Paulo State University – UNESP, Botucatu, Brazil; c College of Veterinary Medicine, Western University of Health Sciences, Pomona, CA, USA

**Keywords:** Dog, canine, intrapelvic mass, hepatoid gland carcinoma, vimentin, E-cadherin

A 14-year-old Teckel dog was presented to a private veterinary hospital with a history of dyschezia, tenesmus and dysuria. Evaluation of the patient's medical records showed that the patient had a history of hepatoid gland adenoma, which had been treated with surgery and orchiectomy 6 years prior. Previous histopathological analysis revealed tumour cells in the trabeculae and cords subdivided by a well vascularized connective tissue stroma. A small population of basophilic reserve cells were found at the periphery of the tumour lobules. Mitotic figures were not observed.

The dog was alert upon presentation to the hospital. Rectal palpation revealed the presence of a large intrapelvic mass. The perineal region showed no abnormalities or signs of atypical growth. A routine complete blood count and serum biochemistry panel were performed, neither of which showed any abnormalities. There was no evidence of lung metastasis on a three-view thoracic radiograph. An abdominal ultrasound identified a large mass located caudal to the bladder, towards the pelvic canal. Cystic areas and foci of calcification were also noted within the mass. Doppler analysis showed moderate vascularization distributed in the periphery of the tumour. There was no evidence of the involvement of other abdominal organs. Ultrasound-guided cytology was performed in the intrapelvic mass and identified a large number of cuboidal-to-columnar cells arranged in clusters. These cells displayed a high nucleus-to-cytoplasm ratio with abundant granular cytoplasm. The nuclei were uniformly round and centrally located and displayed reticular chromatin and a single nucleolus, which are features suggestive of a carcinoma.

**Figure 1.**
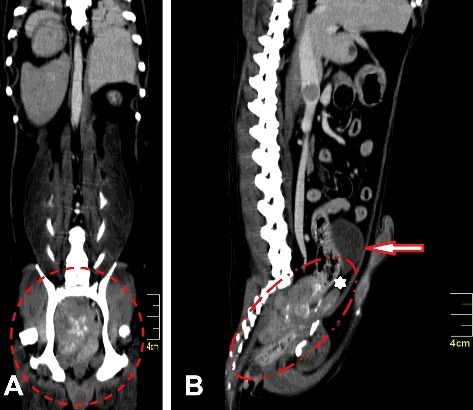
(A) Intra-pelvic tumour in a 14-year-old Teckel dog occupying the entire pelvic canal (dotted circle). (B) Tumour was located caudal to the bladder (arrow), towards the pelvic canal and was adherent to the prostate gland (asterisk).

The patient was referred for computed tomography (CT) to determine the degree of tumour infiltration and assess the possibility for surgical resection (Figure 1(A)). CT revealed that the tumour was located caudal to the bladder, towards the pelvic canal and was adherent to the prostate gland (Figure 1(B)). The tumour had invaded the rectum, adjacent musculature, caudal vena cava and pelvic bones. We evaluated the two anal sacs and found no abnormalities. As complete surgical resection was not possible, a laparotomy was performed to obtain a biopsy and make a histopathological diagnosis. An unresectable, infiltrative and irregular mass showing necrotic areas was identified.

**Figure 2.**
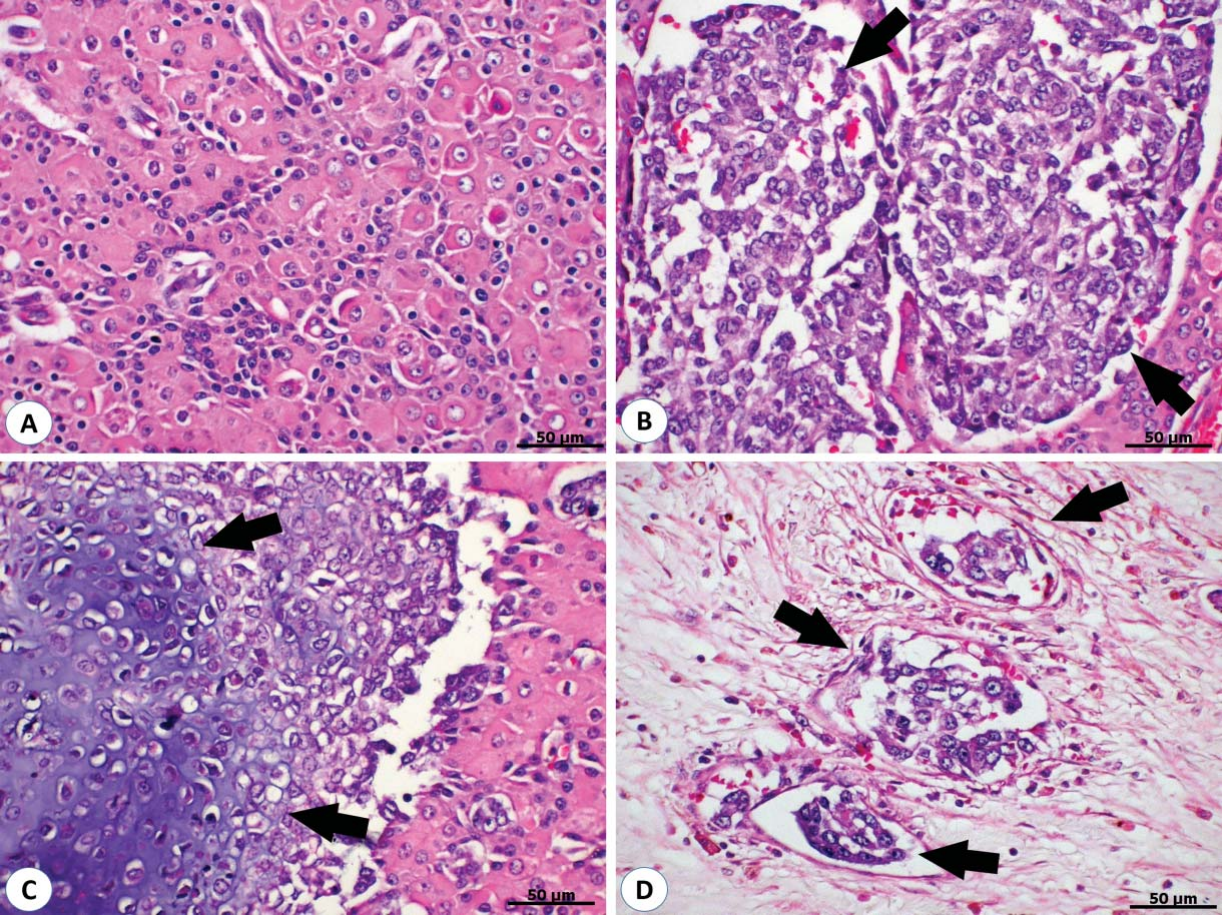
Intrapelvic hepatoid gland carcinoma of the 14-year-old Teckel dog. (A) Haematoxylin and eosin analysis of a primary intra-pelvic tumour. Note the well-differentiated cell population of hepatoid cells characterized by ample cytoplasm, eosinophilic granules and vacuoles, and round nuclei with coarse chromatin. The reserve cells are smaller and have an irregular distribution. (B) Undifferentiated cell population (black arrows) composed by cells organized in nests in a solid pattern with indistinct cell borders, round vesicular nuclei and multiple nucleoli. (C) Multifocal areas of cartilaginous metaplasia (well-formed hyaline cartilage) surrounded by undifferentiated cells (black arrows). (D) Blood vessel (black arrows) invasion by neoplastic undifferentiated cells. 400×.

Histopathological analysis revealed the presence of a fibrovascular capsule and cells within lobules forming a solid pattern. The tumour possessed characteristics of a well-differentiated carcinoma and contained undifferentiated areas. The differentiated region was composed of two cell types: hepatoid and reserve cells (Figure 2(A)). The hepatoid cells were characterized by ample cytoplasm, eosinophilic granules and vacuoles, and round nuclei with coarse chromatin and one or two prominent nucleoli. The reserve cells were smaller, basophilic and had hyperchromatic nuclei with irregular distribution. The undifferentiated area was composed of cells organized in nests to form a solid pattern, indistinct cell borders, round vesicular nuclei and multiple nucleoli (Figure 2(B)). Multifocal areas of cartilaginous metaplasia (well-differentiated hyaline cartilage) were observed in the centre of these cells (Figure 2(C)). Occasionally, the undifferentiated cells were observed in lymph vasculature and blood vessels (Figure 2(D)). An average of 23 mitotic figures were noted in ten higher-power fields (400×). In addition, moderate anisocytosis and anisokaryosis were observed. Multifocal areas of the coalescent necrosis were associated with calcification, and moderate amounts of cholesterol clefts and haemosiderosis were identified.

The results of the histopathological evaluation were suggestive of a hepatoid gland carcinoma. Due to the presence of the two aforementioned cell types, we performed immunohistochemistry staining against pan-cytokeratin (AE1/AE3, Thermo Fischer Scientific, Waltham, MA, USA) at a 1:100 dilution, high-molecular-weight cytokeratin (HMWC) (Dakocytomation, Carpinteria, CA, USA) at a 1:50 dilution, E-cadherin (Dakocytomation, Carpinteria, CA, USA) at a 1:200 dilution and Vimentin (V3, Thermo Fischer Scientific, Waltham, Massachusetts, USA) at a 1:400 dilution. The slides containing the sections were dewaxed in xylol and rehydrated in graded ethanol. For antigen retrieval, slides were incubated in citrate buffer (pH 6.0) in a pressure cooker (Pascal®; Dako, Carpinteria, CA, USA) for 30 s before being treated with freshly prepared 3% hydrogen peroxide in methanol for 20 min to inhibit endogenous peroxidase activity. Then, slides were washed in Tris-buffered saline. A polymer system (Envision, Dako, Carpinteria, CA, USA) was applied as the secondary antibody conjugated to peroxidase and 3′-diaminobenzidine tetrahydrochloride (DAB+, Dako, Carpinteria, CA, USA) as the chromogen for a 5-min incubation, after which Harris haematoxylin counterstaining was performed. Slides were rinsed with Tris-buffered saline after each step of the staining process. Negative controls of each antibody were generated using mouse isotype-specific immunoglobulins (Universal Negative Control, Mouse – Dako, Carpinteria, CA, USA). Normal canine prostate was used as a positive control for epithelial markers.

**Figure 3.**
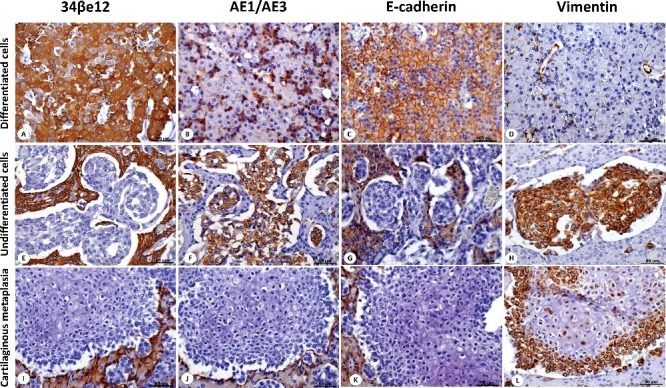
Intrapelvic hepatoid gland carcinoma of the 14-year-old Teckel dog. (A) High molecular weight cytokeratin (HMWC) expression in differentiated cells. Hepatoid and reserve cells display strong membranous and cytoplasmic HMWC expression. (B) Pan-cytokeratin (AE1/AE3) expression of well-differentiated hepatoid and reserve cells. Hepatoid cells show low cytoplasmic expression, and a few reserve cells have moderate cytoplasmic expression. (C) Hepatoid and reserve cells show strong E-cadherin membranous expression. (D) Differentiated cell population was negative for vimentin expression. There are positive blood vessels (brown staining -positive internal control) stained for vimentin. (E) Undifferentiated cells arranged in nests exhibit no HMWC expression (blue staining) and show strong pan-cytokeratin expression (F). The undifferentiated cell population showed no E-cadherin expression (G) and had strong vimentin expression (H). The cartilaginous metaplasia was negative for HMWC (I), pan-cytokeratin (J), E-cadherin (K) and had strong vimentin expression (L). Harris haematoxylin counterstaining, 400×.

The two tumour tissue groups (differentiated versus undifferentiated) exhibited different immunoexpression patterns with respect to the above markers. The differentiated tumour tissue (hepatoid and reserve cells) displayed strong membranous expression of high-molecular-weight cytokeratin (34βE12) (Figure 3(A)), low cytoplasmic expression for pan-cytokeratin (AE1/AE3) (Figure 3(B)), strong membranous E-cadherin expression (Figure 3(C)) and was negative for vimentin staining (Figure 1(D)). The undifferentiated tumour cell population had no high-molecular-weight cytokeratin (34βE12) (Figure 3(E)), showed diffuse positive cytoplasmic expression of pan-cytokeratin (Figure 3(F)), negative E-cadherin expression (Figure 3(G)) and strong vimentin expression (Figure 3(H)). The undifferentiated cells surrounding the well-differentiated hyaline cartilage were negative for high-molecular-weight cytokeratin (Figure 3(I)), pan-cytokeratin (Figure 3(J)) and E-cadherin (Figure 3(K)), with strong vimentin expression. Neoplastic cells were observed invading lymph vessels. These cells stained positively for vimentin and pan-cytokeratin and negatively for HMWC and E-cadherin.

The veterinary literature recommends treating invasive hepatoid gland carcinoma with aggressive surgical removal with adequate margins combined with radiotherapy (Turek and Withrow 2013). However, CT demonstrated that complete surgical resection of the mass was not possible in our patient. Therefore, metronomic chemotherapy was administered, as recommended in a previous study (Fonseca-Alves et al. 2015). The protocol was based on dual administration of piroxicam (0.3 mg/kg BW/day) and cyclophosphamide (10 mg/m^2^/day). The animal showed no signs of intolerance to metronomic chemotherapy. After three weeks of treatment, the patient showed remission of the clinical signs of disease (dysuria, dyschezia, tenesmus). After the patient had received cyclophosphamide for three consecutive months, the dosage was adapted such that the medication was administered every 48 h. After seven months of treatment, the animal presented with dyspnoea and anorexia. Metronomic chemotherapy was suspended, and a 3-view thoracic radiographic examination was performed, at which time the presence of nodules was observed, which is a finding suggestive of metastasis. Support treatment was administered with 2 mg/kg BW of ranitidine every 12 h and 0.3 mg/kg BW of metoclopramide every 8 h, and feeding recommendations for the patient included commercial food with a higher percentage of protein. The patient died four days later, and the owner refused a postmortem examination.

With the exception of prostate cancer, primary intrapelvic tumours are extremely rare in veterinary medicine. Previous studies have described chondrolipomas, chondrosarcomas, leiomyosarcomas, haemangiosarcomas and iliac metastasis from anal sac carcinomas (Mutinelli et al. 2007; Spector et al. 2011). Based on the location of the tumour, as well as the clinical signs displayed by the patient and the CT imaging and cytology results, the presumptive diagnosis was carcinoma of undetermined origin. However, the results of the histopathologic analysis indicated that the diagnosis was hepatoid gland carcinoma. The literature features few studies of hepatoid gland carcinoma causing intrapelvic lymph node metastasis and pelvic obstruction (Vail et al. 1990; Turek and Withrow 2013). Our patient had a history of hepatoid gland adenoma treated with surgical excision and castration.

Perianal gland tumours are common in dogs. The majority of these tumours are adenomas arising from the hepatoid glands (Mutinelli et al. 2007). Hepatoid gland carcinomas are malignant tumours arising from the perianal region that show more invasive behaviour than adenomas (Turek and Withrow 2013). The gross appearances of canine hepatoid gland adenomas and carcinomas are similar; thus, the microscopic morphologies of each tumour are important for their diagnosis (Jakab et al. 2009). Microscopically, well-differentiated perianal gland carcinomas show a similar morphology and architecture to perianal adenomas. As such, detailed analysis of infiltration at the periphery of the neoplasm, irregular distribution of basaloid reserve cells and the presence of mitotic figure in the well-differentiated hepatoid cells might indicate malignancy (Goldschmidt and Hendrick 2002). At the time of tumour (hepatoid adenoma) diagnosis, an abdominal ultrasound examination was performed, and no abnormalities were detected. Hepatoid adenoma has an excellent prognosis. The recurrence rate of the disease is less than 10%, and hepatoid gland carcinoma is less common following castration (Goldschmidt and Hendrick 2002). Regarding our patient, a large amount of time elapsed between the point at which the previous perineal hepatoid gland adenoma was treated and the new intrapelvic hepatoid carcinoma was diagnosed (six years). In addition, CT showed the intrapelvic hepatoid carcinoma did not involve the perineal region. As no perianal mass was present, it was hypothesized that the patient had a primary hepatoid gland carcinoma that invaded the intrapelvic region. An ectopic hepatoid gland in the pelvic canal may explain the development of such a carcinoma.

The results of the histopathological evaluation suggested that two distinct tumour cell groups were present: differentiated and undifferentiated cells. These cells likely shared the same origin but exhibited different degrees of differentiation. The undifferentiated tumour cells showed loss of E-cadherin and HMWC and were positive for vimentin and pan-cytokeratin expression. Furthermore, these cells exhibited fusiform morphology. Thus, the immunohistochemistry results were suggestive of epithelial-mesenchymal transition, a phenomenon that was observed in the lymphatic emboli (all cells invading lymph vessels were HMWC-/E-cadherin-/Pan-cytokeratin+/vimentin+). Previous studies have shown expression of basal markers (Vos et al. 1993) and absence of low molecular weight cytokeratin expression (Kato et al. 2007) in normal hepatoid cells (reserve and differentiated hepatoid cells) and in hepatoid tumours. Vos et al. (1993) reported positive vimentin expression in perianal gland tumours; however, these authors had a heterogeneous tumour group (moderately and poor-differentiated perianal tumours) with different histological subtypes (anal sac carcinomas and hepatoid gland tumours). Thus, it was not possible to correlate vimentin expression with hepatoid gland tumours. Recently, Pieper et al. (2015) evaluated vimentin expression in a unique case of well-differentiated canine hepatoid carcinoma with positive vimentin expression and suggested EMT was occurring in this tumour. On the other hand, despite the positive vimentin expression, the tumour from this study had no population of poorly differentiated cells (Pieper et al. 2015).

In summary, a hepatoid carcinoma was reported displaying pelvic canal invasion rather than the classic perianal presentation. Metronomic chemotherapy was effective in reducing the patient's clinical signs and improved the patient's quality of life.
